# Home deliveries in the capital: a qualitative exploration of barriers to institutional deliveries in peri-urban areas of Lusaka, Zambia

**DOI:** 10.1186/s12884-018-1837-7

**Published:** 2018-06-01

**Authors:** Tamara Mulenga, Misinzo Moono, Martha Mwendafilumba, Albert Manasyan, Anjali Sharma

**Affiliations:** 1 Centre For Infectious Diseases Research In Zambia (CIDRZ), P.O. Box 34681 Plot 34620 Off Alick Nkhata Road, Mass Media, 10101 Lusaka, Zambia; 2 University of Alabama at Birmingham, Birmingham, USA; 3 University of Washington, Seattle, USA

**Keywords:** Low-middle income countries, Maternal health, Child birth, Respectful maternity care, Zambia, African traditional medicine, Midwifery

## Abstract

**Background:**

A shortage of skilled birth attendants and low quality of care in health facilities along with unattended home deliveries contribute to the high maternal and neonatal mortality in sub Saharan Africa. Identifying and addressing context-specific reasons for not delivering at health care facilities could increase births assisted by skilled attendants who, if required, can provide life-saving interventions.

**Methods:**

We conducted 22 in-depth interviews (IDIs) with midwives at three health facilities in peri-urban communities and 24 semi-structured surveys with mothers in two areas served by health facilities with the highest number of reported home deliveries in Lusaka, Zambia. Both IDIs and surveys were audio-recorded, transcribed and coded to identify themes around delivery and birthing experience.

**Results:**

We found that most women preferred institutional deliveries to home deliveries, but were unable to utilize these services due to inability to recognize labour symptoms or lack of resources. Midwives speculated that women used herbal concoctions to reduce the duration of delivery with the result that women either did not present in time or endangered themselves and the baby with powerful contractions and precipitous labour. Respondents suggested that disrespectful and abusive maternity care dissuaded some women from delivering at health facilities. However, some midwives viewed such tactics as necessary to ensure women followed instructions and successfully delivered live babies.

**Conclusion:**

Difference in beliefs and birthing practices between midwives and mothers suggest the need for open dialogue to co-design appropriate interventions to increase facility usage. Further examination of the pharmaceutical properties and safety of herbal concoctions being used to shorten labour are required. Measures to reduce the economic burden of care seeking within this environment, increase respectful and patient-centred care, and improve the quality of midwifery could increase institutional deliveries.

**Electronic supplementary material:**

The online version of this article (10.1186/s12884-018-1837-7) contains supplementary material, which is available to authorized users.

## Background

Of the approximately 303,000 maternal deaths recorded in 2015 worldwide, the largest number occurred in sub-Saharan Africa (SSA) with an estimated 201,000 deaths (66.3%) [1, 2]. Additionally, preterm birth complications and intrapartum-related events took approximately 694,000 neonatal lives in SSA in that same year. Only ten countries have achieved Millennium Development Goal (MDG) 5 which calls for a 75% reduction in maternal mortality ratio between 1990 and 2015 [3]. Moreover, over five times more deaths still occur within 24 h than at any other age among under 5 s (MDG 4) in many low and middle-income countries (LMIC) [3, 4]. Approximately one-third of early neonatal mortality in countries with the highest rates of neonatal mortality were intrapartum-related and half of those births occurred at home without skilled birth attendants [4, 5]. Reasons for low uptake of facility deliveries in SSA include long distances to facilities, lack of transport, poor quality of care related to staff shortages and poor attitudes of healthcare providers, education level of mothers and cultural beliefs [6, 7]. Identifying and addressing context-specific reasons for not delivering at health facilities could increase births assisted by skilled attendants who, if required, can provide life-saving interventions [8].

In 2015, the maternal mortality ratio in Zambia was 225 per 100,000 live births, 3 times higher than Sustainable Development Goal 5 (70/100000) [9]. In the last 20 years, the Zambian government has encouraged women to give birth in health facilities by removing user fees for maternal and child-health services and banning Traditional Birth Attendants (TBAs) in order to discourage home deliveries [10, 11]. Additionally, in 2002, Safe Motherhood Action Groups (SMAG) were introduced across the country [12]. SMAGs, groups of community based volunteers, encourage women to deliver at health facilities and educate communities on pregnancy, childbirth and breastfeeding related topics [13]. Nonetheless, between 1992 and 2014, the percentage of facility deliveries in Zambia increased by only 16% from 48 to 64% for the reasons mentioned above and poor staff retention particularly in rural areas [9]. Little is known about who attends to women during home births since TBAs were made illegal.

Barriers to uptake of facility deliveries in Zambia are under-studied, particularly for urban areas where the last study was conducted in 2003 [14]. Lusaka’s urban population increased by 5% per annum between 2000 and 2010 with most urban growth occurring in Lusaka city and this is predicted to continue [15]. It is therefore important to understand both providers’ and mothers’ perspectives on barriers to uptake of facility deliveries within peri-urban areas, which is precisely what this study aimed to do [16, 17]. Additionally, this study sought to compare behavioural drivers for facility delivery among both women who did and did not deliver a baby in health facilities in the last two years. These insights into barriers and drivers of facility delivery will help the Zambian government and relevant stakeholders formulate a more effective strategy to increase facility deliveries and reduce maternal and neonatal deaths.

## Methods

This qualitative study draws on semi-structured in-depth interviews (IDIs) with midwives and a semi-structured survey with mothers to explore how individual’s views, cultural teachings, experiences and perceptions affect the utilisation of healthcare facilities for delivery. Additionally, we explored the influence of midwives’ experiences and conduct during labour and pregnancy, both in health facility settings and in their personal lives.

### Study setting

Lusaka city, the capital of Zambia, is predominantly urban. Lusaka has the largest population among all districts in Zambia with an estimated population of 1,854,907 according to the last census [18]. Lusaka province has the lowest fertility rate among the ten provinces in the country at 3.7 births per woman and is relatively wealthier [19]. Public health facilities in Lusaka operate several outreach initiatives in conjunction with SMAG volunteers. Female relatives accompany women to the clinic but are not allowed in the labour ward. Midwives conduct facility-based deliveries, referring complicated cases to doctors. Of the 193 health facilities in Lusaka, only 15 (both private and public) offer delivery services within Lusaka city [20]. Currently, the average percentage of facility deliveries within Zambia is 67% for both rural and urban areas, and above average at 89% for Lusaka province [19].

This study was conducted as part of the Preterm Resources, Education and Effective Management for Infants (PREEMI) programme implemented to improve the quality of maternal and child health services in Lusaka district between 2014 and 2017. PREEMI was implemented in three peri-urban health facilities in Lusaka city, namely Chawama Hospital, Chipata Hospital and George Clinic. The hospitals are designated as First Level Hospitals, the equivalent of District hospitals which are defined by the Zambian government as referral hospitals found in all districts that are intended to serve a population of between 80,000 and 200,000 [20].

IDIs were conducted in the three peri-urban Lusaka health facilities served by the PREEMI program. These health facilities provide antenatal, post-natal, delivery, basic or comprehensive emergency obstetric and neonatal care (EmONC), among other services. Most women within the peri-urban areas of Chawama, George and Chipata ‘compounds’ (informal settlements) deliver at their local health facilities, which are within a 5 km radius [21]. However, the PREEMI project received persistent reports of home deliveries occurring within these areas and particularly within Chipata Hospital’s catchment area. Two zones (Zone 8 [Kabanana, approximately 4 km away from Chipata Hospital] and Zone 2 [Maziopa, approximately 2 km away from Chipata Hospital]) were purposively selected as survey sites because they had the highest number of home deliveries among the Chipata Hospital catchment zones.

### Sampling

A convenience sample of 24 mothers, who met the inclusion criteria of being resident in Zone 2 and Zone 8 of Chipata catchment area for at least 3 months and who had delivered a child within the catchment area in the last two years, participated in the survey. Convenience sampling is a non-random sampling technique whereby convenient cases who meet the required criteria are located and selected on a first-come-first-served basis until the sample size quotient is full [22]. Participants who were below 18 years old could participate if they gave consent together with their guardian. All eligible participants were conversant in either Bemba, Nyanja or English.

Twenty-two qualified midwives working within the Labour or Maternal and Child Health Wards at the three facilities were recruited for IDIs using convenience and snowball sampling to accommodate the shift-work and staff shortages at all facilities. Snowball sampling, where participants recommended colleagues who might meet the inclusion criteria, who in turn referred other colleagues, allowed the researchers to access midwives who would have been otherwise excluded because they worked at night or were absent on the day of interview [23]. All participants were identified, recruited and enrolled during the same week they took part in the study. All the midwives were conversant in English and aged 18 years old and above.

### Data collection

Two Zambian Research Fellows and one Research Assistant conducted IDIs with midwives in English, the language of practice. All IDI study team members possessed at least a diploma (a non-university tertiary education qualification in any subject) and had varying levels of experience conducting qualitative research. They underwent Human Subjects Protection (HSP) training and IDI refresher training before data collection. One-on-one IDIs were conducted in a private, quiet room in the three health facilities and were audio recorded following written consent. The interview guide (see Additional file 1) developed based on published literature on traditional birthing beliefs, disrespect and abuse and determinants of facility usage for delivery in SSA. This was then reviewed by senior supervisors. Examples of questions include: “*How was what you learned in your training different from what is taught traditionally*?”; and “*How would you describe your delivery experience for your last child?”.* Disrespect and abuse were defined as any references to shouting, hitting, purposeful neglect or use of demeaning language towards women delivering in the labour ward [24, 25]. For these interviews, the terms ‘cultural teachings’ and ‘traditional teachings’ were used interchangeably and defined as any teachings or practices held in common by a group of people that originate from the same tribe or similar social group [26]. One of the Research Fellows conducted debriefing sessions with other data collectors at the end of each day and information from these sessions were used to decide which topics should be further probed during the next IDIs.

The interviews with mothers were conducted using a semi-structured questionnaire (see Additional file 2) adapted from the close-ended Barrier Analysis Survey for behaviour change tool to include some open-ended questions to explore topics in greater depth and to ensure freely given responses by removing judgemental categories of ‘doers’ and ‘non doers’ [27]. The questionnaire collected information on perceived advantages and disadvantages of using health facilities for delivery, expressed knowledge of possible labour complications and the influence of participants’ social circles on the decision to use a health facility for delivery. All questionnaires were translated into Bemba and Nyanja to allow participants to decide in which language they preferred to be interviewed. Data collectors comprised of eight women, three of whom were involved in the IDIs and one of whom worked at Chipata Hospital as Community Liaison Officer under the PREEMI programme. The other four members of the team were SMAG volunteers from Zones 2 and 8, familiar with women in the hospital catchment areas. SMAG volunteers were paired with the other study team members, all of whom underwent HSP and data collection training prior to data collection. Two pairs of data collectors were allocated to each zone to avoid duplication. Each pair interviewed four mothers who had delivered at Chipata Hospital and two mothers who had delivered at home. Both illiterate and literate mothers with varying levels of educational attainment participated in the survey. The survey was conducted in each participant’s home, preferably in a quiet place. To improve the accuracy of responses recorded on data forms, all interviews were audio-recorded. Of the mothers recruited, two were under 18 years old and participated with their guardians’ consent.

### Data analysis

Thematic analysis was used to systematically identify and code recurring patterns within the textual data to describe social reality around birthing experience and the meaning that both women and midwives assigned to it as described below [28]. Research assistants transcribed the audio recording of each interview directly into English, of which the first two authors reviewed a sample for accuracy. Research Assistants and authors discussed the most appropriate English translation for words that did not have direct English equivalents. Research Assistants transcribed both interviews and responses to open-ended questions in the survey centrally onto a secure, password-protected laptop. The first two and the last authors coded the first three IDI transcripts using inductive and deductive reasoning to identify emerging themes [29]. The codes and their use were standardized and the code book finalized through this initial coding of transcripts. Survey responses were deductively coded using pre-existing codes in the literature and the original Barrier Analysis Survey. The first two authors manually coded IDIs and textual data from the survey using Microsoft Word [30]. Responses to close-ended survey questions were subjected to descriptive and comparative analysis in Excel.

Themes were created by converting units of analysis, extracted from interview text, into codes, sub-themes and eventually themes through a stepwise process. Table 1 illustrates how relevant sections of text were extracted as meaning units and summarised into condensed meaning units to create codes.

**Table 1:** Examples of meaning units, condensed meaning units, sub-themes and themes from thematic analysis of interviews with midwives

Meaning unit	Condensed meaning unit	Code	Sub-theme	Theme
*“like in some traditions, people are restricted on what to eat; some are not allowed to eat eggs which is not good…and others are told when they deliver them, they should not leave their placenta at the hospital*”	Some traditions have specific food and delivery restrictions	restriction	Prescribed behaviour	Traditional practices and teachings about labour
*“you do not need to have sex with other men when you are married or when you are pregnant.... you can die whilst giving birth when you have sex with more than one man*”	Belief that infidelity can lead to death in labour	Sex and pregnancy myths		
*“they are told to drink something, ok the African syntocinon which they so much believe in that it works and the woman who doesn’t drink African syntocinon may stay in labour for more than 3 days”*	Given herbal concoction to speed up labour	Going against nature	Traditional interventions	
*“some women are also taught or they believe that they should take something to accelerate labour, so that when they come to deliver their labour is quicker they don’t stay a longer period in that painful period [of labour] but then at the end of the day most of them end up having precipitated labour… that has its own implications”*	Traditional interventions can lead to delivery complications	Negative outcomes		

The two researchers discussed and contrasted the various codes before further categorising them into sub-themes based on similarities between underlying meanings. Finally, similarities between the overarching message of the sub-themes was used to develop themes. Relevant themes have been clustered into three overriding themes that illustrate that behaviours around labour and delivery for both parties are driven by cultural influences that either compete or are utilised alongside medically driven reasoning and individual characteristics.

## Results

### Participant characteristics

A total of 46 participants took part: 22 midwives in IDIs and 24 mothers in the survey (Tables 2 and 3). Twelve mothers, four of whom had home deliveries, were recruited from each zone. Data from one mother from ‘Zone 2’ was excluded as she did not meet the inclusion criteria.

**Table 2:** Midwife demographic details

	Midwives
Characteristic	N = Number
Age	
20–30	5
30–40	9
40–50	5
50–60	2
60–70	1
Midwifery training	
College trained	18
University trained	4
Marital status	
Single	2
In a relationship	1
Married	17
Divorced	1
Widowed	1
Religion	
Christian	22
Length of stay with current health facility	
< 1 year	4
1–5 years	11
6–10 years	6
> 10 years	1
Total	22

**Table 3:** Socio-demographic details for mothers enrolled in the survey

Mothers	Zone 2 *N* = 12	Zone 8 *N* = 11	Total *N* = 23
Age			
< 25	6	2	8
25–34	2	7	9
35–44	4	1	5
45–54	0	1	1
Marital status			
Single	2	0	2
Married	9	11	21
Tribe			
Bemba	4	2	6
Chewa	6	2	9
Tumbuka	1	1	2
Shona/Ngoni	1	0	1
Namwanga	0	2	2
Mambwe	0	3	3
Tonga	0	1	1
Occupation			
Unemployed	9	6	15
Business woman	2	3	5
Cleaner	1	0	1
Tailor	0	1	1
Student	0	1	1
Number of children			
1	5	2	7
2	2	2	4
3	2	3	5
4	0	2	2
5	1	1	2
> 5	1	1	2
Age at first child^a^			
< 18	4	1	6
18–25	7	7	14
Place of delivery			
Chipata First Level Hospital	8	8	16
Home	4	3	7

^a^One participant from Zone 2 and 3 participants from Zone 8 missing details

Among the 22 midwives recruited for the IDIs, eight were recruited from Chipata Hospital and George Clinic and six from Chawama Hospital. On average midwives were 36.5 years old, Christian and approximately three quarters were married. One midwife was male. Four midwives were trained to university degree level while the rest were trained to college level.

The average age for mothers from both zones fell within the 25–34 age bracket and only two were married. Most mothers from both zones were unemployed while a quarter of women in Zone 2 and over a third of women in Zone 8 were either employed or enrolled as a student.

Women’s education levels were not noted. Literacy levels were assessed by asking women to read one paragraph of the consent form aloud in one of the three languages available (English, Bemba or Nyanja). Four women were unable to read the consent forms in any of these languages and were classified as illiterate.

### Themes and sub themes

The three main themes that emerged included traditional beliefs, medical focus on live birth, and individual characteristics/differences. Sub-themes on traditional beliefs were related to maternal and child health, conjugal relations and labour and delivery rituals, all of which influenced women’s practices and midwives’ treatment of women. Overwhelming focus on live births rather than maternal comfort coupled with staff shortages led to disrespectful and abusive behaviour by midwives. Women’s socio-economic status, literacy levels, experience of labour, and interaction with female elders were offered as explanations for individual differences in seeking institutional deliveries.

### Impact of traditional beliefs on pregnancy and labour

Midwives revealed that they encountered a multitude of traditional beliefs and practices both among pregnant and birthing women as well as in their personal lives. The midwives alluded to elders, specifically grandmothers, being the primary source of traditional teachings and enforcers of these practices. Both midwives and mothers reported that women are initially exposed to traditional beliefs and practices at the start of puberty. Teachings continue during preparation for marriage and with each pregnancy in increasing levels of detail on labour and delivery. However, information may vary as teachings are given “*per individual*” and “*in secrecy*”. As many residents within peri-urban areas hail from different parts of the country, midwives also revealed that the diverse cultural backgrounds of their clients are reflected in the variety of traditional practices witnessed:

“*I will give a perfect example of women I have delivered so far from this community, the Ndebeles [Zimbabwean tribe], those who stay in Malapodi. When you have such a woman who comes in labour, you are going to have a proper delivery because for them they are taught that ‘labour is a very painful process and you have to bear with it until the child is born. If there are complications the people at the hospital will help you identify them and they will solve the problem.’ [However,] there were some people who came from Eastern Province and their knowledge is not like what we are taught at school. I heard that if someone is in labour and they want them to deliver they would dig a hole and then they would put something like a cloth down then they would ask that woman to squat there and make them starting pushing [the baby].*” **(IDI, Health Facility A, Midwife 5)**

Thus, midwives thought that some tribes birthing practices align with modern midwifery while others do not fit within the medical model of labour and delivery. However, there was no indication that tribal or regional rivalries played a role in how midwives treated women or perceived their behaviour during the birthing process. Many midwives reported that they actively counteract traditional beliefs and practices through sensitisation in the communities as well as during labour and delivery with variable impact.

Women living within peri-urban communities were exposed to a variety of beliefs and practices other than from their own cultural groups. However, it was unclear from the data whether this exposure had influenced practice. Nonetheless, three distinct categories of traditional beliefs emerged from the interviews with both mothers and midwives related to maternal and child health, conjugal relations, and labour and delivery rituals.

Beliefs related to health and future welfare of the baby and recovery and well-being of women in the post-partum period led to restrictions on women’s diet and movement respectively:

“*Pregnant women are not supposed to eat eggs because the babies will be born without hair.”* **(IDI, Health Facility A, Midwife 3)**

“*When a woman delivers … mmhhm … they are not allowed to cook or help out with those, you know heavy chores [physically tiring] after delivering*.” **(IDI, Health Facility B, Midwife 6)**

The second category of traditional beliefs were those related to conjugal relationships. The advice based on traditional beliefs around sex could be contrary but we were unable to understand the underlying reasons, such as tribal or regional affiliations. For instance, as the two quotes below show, women could be told to continue or to stop having conjugal relations for the sake of the baby’s health:

“*They say you should continue meeting [having sexual intercourse with] your husband until the baby is born to strengthen the baby*.” **(IDI, Health Facility A, Midwife 2)**

*“When you are about to deliver, you have to like stop making love with your husband. There are such beliefs. They believe that if they continue doing that, the baby will be born with the semen … The sperm for the husband, it will just be there on the baby’s body and all that.”* **(IDI, Health Facility C, Midwife 2)**

Sexual dalliances by pregnant women and their sexual partners were presented as having short and long-term impact on the baby. Extra-marital affairs were presented as deadly to women as they are believed to result in certain death during labour and deliver as described below:

“*When you are pregnant, you are not supposed to sleep with any other guy and all that, any other man. They believe if you do that, you may die on the delivery bed.”* **(IDI, Health Facility C, Midwife 2)**

Some mothers also revealed that complications during labour were sometimes attributed to supernatural causes including charms and curses used by the husband’s mistresses to ensure the wife did not have successful labour:

*“if she is bewitched … she cannot manage to deliver the baby but she will feel the pain.”* **(Zone 2, Mother 8)**

Some of the midwives corroborated that this belief led to the use of traditional remedies to ward off charms used by husband’s mistress to avoid labour complications:

*“Some women even drink some things, some herbal medicines. They will tell you, ‘No, maybe my husband had a girlfriend so I took this so that I don’t find myself in any complications [during l abour].”* **(IDI, Health Facility A, Midwife 3)**

Some midwives praised beliefs that restricted extra marital affairs for both men and women because of decreased risk of HIV acquisition. The midwife from Health Facility C explained that “In *Bemba, sometimes they call it “Inchilla” …”* and this belief is encouraged by the midwives because it deters pregnant women and their partners from engaging in extramarital affairs.

More commonly, however, there was an overwhelming condemnation of traditional beliefs and practices among the midwives because of the perceived negative impact they have on delivery outcomes:

“*It [traditional practices] is really giving us a very negative, negative outcome because even the babies when they are born, sometimes they are asphyxiated…”.* **(IDI, Health Facility A, Midwife 1)**

According to both midwives and mothers, grandmothers and female elders gave mothers in labour “*African syntocinon*”, a herbal concoction believed to hasten delivery and compromise women’s ability to seek care in a timely manner**:**

“*Some relatives prefer tradition, when you are in labour, they can give you herbal medicine to take and within minutes you would have delivered*”. **(Zone 8, Mother 1)**

Midwives hypothesised that the *African Syntocinon* did hasten labour and thus, contributed to high numbers of home deliveries, occurrence of foetal distress and some cases of post-partum haemorrhage:

“*Even the African syntocinon, the herbal concoction that they drink in order to help them deliver quickly … When a woman is given such concoctions, they normally deliver placentas that are a bit greenish … Most of the babies that I have seen from women who have confessed to have drunk such traditional medicine, we have to resuscitate the babies at birth … It will give a woman such strong contractions, such that a woman may even have a ruptured uterus* …”. **(IDI, Health Facility B, Midwife 3)**

The secrecy around *African Syntocinon* intake added to the challenges posed to conducting safe deliveries because midwives would prefer to monitor women who have taken herbal concoctions more closely than other pregnant women for unexpected complications from an accelerated labour:

“A*m more vigilant because I don’t know to what extent this medicine is going to affect [the delivery]. So am more vigilant on that mother … mmhh … who has taken the medicine*”. **(IDI, Health Facility A, participant 5)**

### Impact of medical focus on live birth rather than maternal comfort

#### Disrespect and abuse

Both midwives and mothers reported that the disrespect and abuse faced by women during labour posed a barrier to facility delivery. Many midwives confessed that the treatment of women at health facilities during the labour period was occasionally “*harsh*”, because their daily experiences and operational context were not conducive to allowing maternal autonomy and empowerment as advocated during their training. Midwives justified harsh words and actions due to the priority given to delivering “*a live baby*” and not to the mothers’ labour experience. For instance, they contend that a woman in labour can get so caught up in their pain that the only way to turn their attention to the midwife’s good medical advice is by scolding or hitting to ensure a live birth:

“*When a woman comes in labour, because of that pain she has, she becomes wild! You know! You can do anything! How can someone jump on a chair?! Jumps down, on the bed…. You try to … so you find she becomes irritable … you try to talk to her in a soft way, she won’t understand. You have to be harsh to her in order for her to get your words.*” **(IDI, Health Facility A, Midwife 1)**

*“The only time that we beat and become aggressive is when we see a woman in labour closes her legs when a baby is about to come out, putting her child in danger. So, we have no option but to beat.”* **(IDI, Health Facility A, Midwife 8)**

*“The reason that nurse was harsh, it’s because she [birthing mother] was not following instructions. Because at the end of the day if, if any, any baby dies in labour, you know a midwife has to write a report and … And there is a form that they have to fill in and no midwife wants to do that!”* **(IDI, Health Facility A, Midwife 5)**

Additionally, some midwives reported that staff shortages contribute towards midwives feeling overwhelmed, which sometimes also resulted in them being disrespectful and abusive towards the mothers:

“*You find the ratio between the nurse and the clients, maybe you attend to so many, maybe 40 or so. One nurse, you are on duty. So, probably that’s what even leads to nurses becoming rude on duty. Maybe it’s the work load that they face!*” **(IDI, Health Facility A, Midwife 6)**

While mothers reported that disrespect and abuse did occur at the health facility, they also appreciated the quality of care offered at medical facilities:

*“When they scold you, it means they are teaching you, so that you can learn.”* (**Survey, Zone 8, Mother 4**)

This positive outlook may be because mothers rely on midwives’ instructions as they do not have access to advice or encouragement from family members or friends who are not allowed into the labour room.

### Impact of individual characteristics/differences

Other than belief systems, many midwives believed low socio-economic status and literacy levels within their catchment area contributed to the practice of home deliveries:

“*With the high class, they believe to say ‘I have to deliver from the hospital,’ including the middle [class]. But with the low [class], for them, it’s either home or hospital, whichever I deliver at, its fine with me.’”* **(IDI, Health Facility C, Midwife 1)**

Midwives insinuated that low socio-economic standing of women was strongly associated with their decision to deliver at a health facility with more affluent women preferring private services. The midwives themselves were of a higher socio-economic status than the women they served, though a few had grown up within these same peri-urban communities. It was unclear whether the socio-economic background of the midwives or the mothers influenced midwives’ treatment of women or their views about the mothers’ behaviours during birthing. However, it is the wealthier among peri-urban residents who make it to the clinic. Mothers reported that economic constraints for some women contributed towards them delivering within their homes even when they intended to deliver at the health facility:

“*What leads to delivering at home is suffering -- you don’t have resources.*” **(Survey, Zone 2, Mother 3)**

Participants used the term ‘resources’ to refer to money and the products they are required to purchase and bring to the health facility when they are in labour. Some of these products include a full bottle of bleach, a bucket, a blanket (for the baby), a chitenge (traditional cloth) and a new pair of scissors. Financial circumstances, specifically lack of adequate income, are presented as an important deterrent for women within this community who want to deliver at a health facility. Those without the financial means to pay for transport and buy the items required for delivering at a health facility are forced to choose home delivery:

*“I know the benefits of using the local health facilities. If I have all the requirements like transport and if life is generally okay, then I can go to the health facilities. But, for instance, if I don’t have the resources, then I can deliver at home as I would have no option.”* ***(*** **Survey, Zone 8, Mother 1)**

**Fig. 1:**
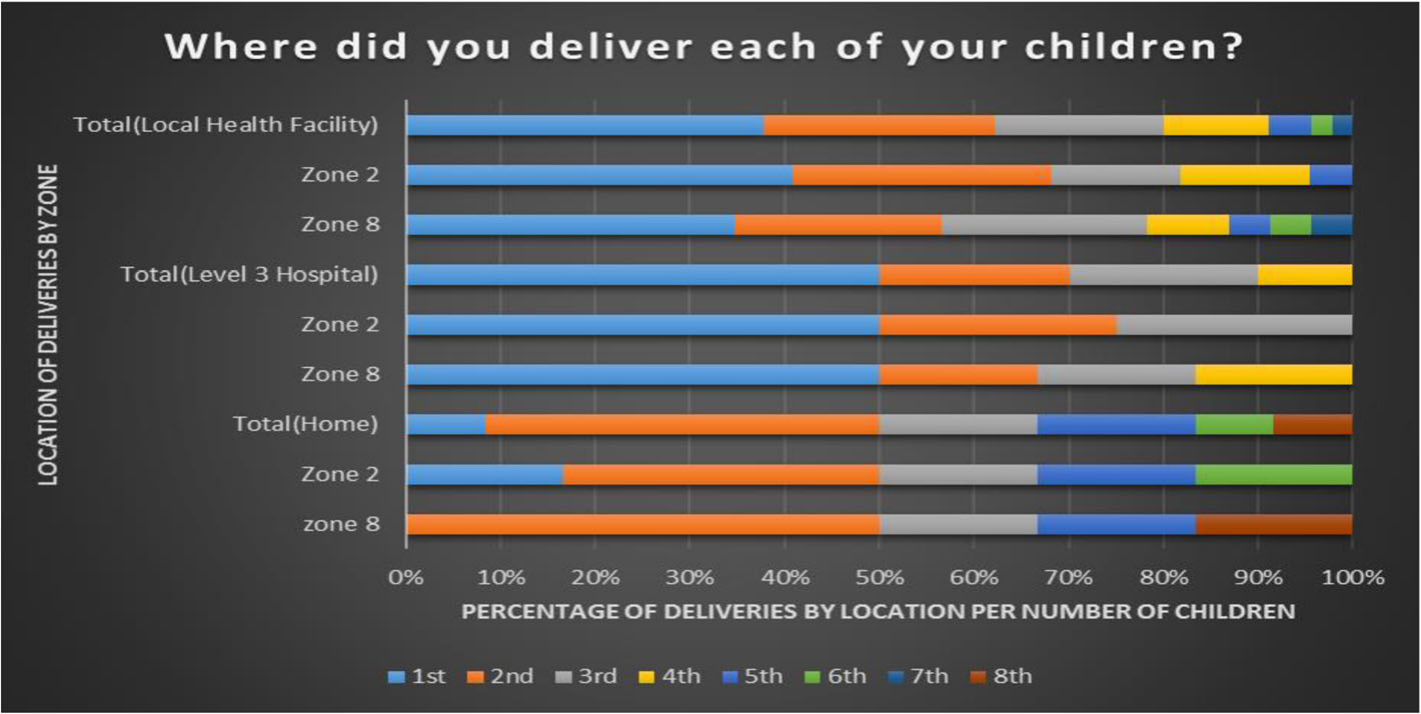
Brief description of the data: Location of deliveries for each child born to all participants comparing Zone 2 and Zone 8

Mothers from Zone 8 were more likely to cite distance and transport constraints as a reason for delivering at home instead of health facilities. Most children in both zones were delivered at their local health facilities or at a Level 3 Tertiary Hospital (Fig. 1). Zone 8 is located 2 km further away from the health facilities in comparison with Zone 2, although the roads in both areas are of the same standard. Figure 1 illustrates that most women in both zones deliver their first children at either a hospital or their local health facility but were more likely to have subsequent deliveries at home.

Other than economic and transport-related constraints, many midwives reported that both nulliparous and multiparous women’s inability to distinguish between the start of labour and pre-labour pregnancy symptoms may lead to home delivery. This view is corroborated by women as illustrated by the quote below where misunderstanding of what labour should feel like, led to home delivery even though they may have preferred to deliver at the health facility:

*“I wanted to deliver at the health facility but because of the same feeling of pain here then there, I was confused. So even when I was delivering, I didn’t think I was in labour at a stage that I could deliver*.” (**Survey, Zone 2, Mother 6**).

Some mothers reported a preference for facility-based delivery being aware of the dangers of delivering within their homes based on experiences within their social networks:

“*Delivering at home is bad! Because, I remember, a friend of mine who had delivered at home. When she delivered at home, then after delivering, the placenta remained in the uterus. It didn’t come out, so like that, and that is a risk*” **(Survey, Zone 2, Mother 1)**

Some women described being misled by elders who wanted them to deliver at home so they could charge the women for their services. It was unclear whether these women were TBAs as some participants noted that “*Some elders in the community [were] home birth attendants*” (Zone 8, Mother 4) while others learnt how to deliver babies in their villages as a rite of passage for all women in their area.

## Discussion

The key behavioural drivers behind the decision to deliver at a health facility or home for mothers in both zones were socio-cultural norms, lack of resources and previous experience of labour. Socio-cultural norms encompassed both long-held traditional beliefs along tribal lines and overwhelmingly, the influence of elderly women irrespective of tribal affiliations who influenced women’s decisions in these peri-urban settings. Lack of monetary and other resources prevented mothers from delivering at health facilities. Women who previously experienced labour and delivery complications preferred institutional deliveries because they trusted in the skills of medical professionals. Apprehension of traditional birth practices, staff shortages and prioritization of live birth over maternal health drove midwives’ behaviour in this setting. Most midwives felt unprepared to deal with some of the popular traditional labour and delivery practices which were contrary to their medical training. Staff shortages further compromised their ability to create a safe environment for deliveries and to provide women with a better experience. These factors, compounded by the fear of having a foetal death, made midwives resort to abusive behaviour and maltreatment to instruct women through their birthing process.

We found that women in our study area preferred to deliver in health care facilities but may deliver at home because they did not realize that they were in labour or could not afford transport. The finding that women could not access care even when available within 5kms of their residence challenges assumptions about distance as a significant barrier to facility deliveries in SSA [31]. An immunisation study carried out in a similar peri-urban community in Lusaka found that people living a little over a kilometre from an immunisation site were less likely to vaccinate their children than those less than half a kilometre away [32]. This suggests that although all women were theoretically closer to health facilities than their rural counterparts, the concept of distance differed in the peri-urban areas identified in this study. We speculate that this was due to lack of financial capital to secure transport or lack of social support to assist in securing transport.

Many mothers in the study were unaware of the variation of labour signs between pregnancies. Although midwives reported success of sensitisation initiatives in improving knowledge about pregnancy, labour and delivery among women in Zambia, women appear to only recognise labour when their signs and symptoms fall within the remit of what has been taught. This finding highlights the need for dialogue between pregnant women and maternity programmes to allow both parties to ascertain how each of them interprets the information that is delivered. This approach is likely to create more effective interventions for increasing facility deliveries that do not cause unintentional harm.

We are the first to report continuation of traditional labour rituals, specifically the use of herbs during labour, among women living in peri-urban Lusaka. Use of traditional herbs to induce labour and reduce its duration has also been documented in South Africa and in Uganda among 80% of their study participants [33, 34] In both countries, many of the herbs commonly used were found to be toxic but without specific information on dosage, the level of their toxicity when used during labour remains unknown [33, 34]. To date, no studies have documented the range of plants used to induce labour in Zambia, their capacity to hasten labour, their toxicity levels or possible impact on unborn children. Although the impact of herbal concoctions on delivery outcomes remains anecdotal, peri-urban and rural health facilities would benefit from further studies that would differentiate what is truly harmful from what is a mere cultural difference. Dialogue between women and health staff regarding traditional beliefs coupled with appropriate sensitisation campaigns that reduce harmful practices will be important as Zambia continues to experience the migration of populations from rural to urban areas [17].

Women’s birthing experience was often sub-optimal because of midwives’ professional interest in ensuring a live birth. Disrespect and abuse towards mothers in labour did not present a significant barrier in this study, unlike that conducted in Ghana [35]. Nonetheless, as reported by other studies, disrespect, abuse and otherwise negative attitudes of midwives towards women seeking delivery services could hamper efforts to increase facility deliveries in peri-urban Lusaka [36, 37]. Our findings suggest that better-staffed health facilities could reduce stress, which has been identified by midwives as a key driver behind their disrespect and abuse towards women seeking delivery services. Providing better management support and quality improvement tools can also help midwives self-regulate their behaviour. An example of this occurred in Ethiopia, where utilisation of the Standards-Based Management and Recognition (SBM-R©) quality improvement tool increased respectful maternity care in health facilities [38]. Findings from this study also highlighted the need for policy makers and the Global Health community to better address poor management of healthcare workers in order to achieve health outcomes which could be applied to other SSA countries [38].

### Strengths and limitations

As a qualitative study, the findings of this study are unique to Lusaka compounds and not generalizable. Our study was limited to the birthing experience in facilities and not at home, so does not explore birth attendants in the home setting and potential incentives for them. While familiarity with the SMAG volunteers involved in data collection may have introduced response bias into the study, other studies show that inhabitants of these compounds prefer to interact with known persons who are familiar with the context within which they live [39]. Also, our study provides in-depth and contextual data on the delivery practices and experiences from both mothers’ and midwives’ perspectives to provide holistic solutions (ones that will address issues beyond those that are purely medical). Similarly, the focus on peri-urban areas is significant given the rapid urbanization of Lusaka along with substantial rural-urban migration which indicate that issues identified in this paper are likely to affect more facilities in the future.

## Conclusion

Overall, this study suggests that increasing institutional deliveries in Zambia, a LMIC with pluralistic health beliefs and significant structural barriers, will require greater dialogue between health professionals and the women they serve to generate relevant and effective interventions. Such interventions would help healthcare workers deal with the variation of beliefs and experiences in a professional and constructive manner and enable women to engage in open dialogue on their labour symptoms, labour-related expectations and their desired birthing experience. Furthermore, we recommend investigations into the reported use of herbal concoctions during labour and delivery in Zambia which, if true, need to be tested for pharmaceutical properties and safety.

## Additional Files

Additional File 1: QIA Midwife Interview Guide Final Quality improvement assessment midwife questions (DOC 40 kb)

Additional File 2: QIA Mother’s Questionnaire Quality improvement assessment mother’s questionnaire v1.0 (DOC 112 kb)

## Abbreviations

EmONC: Emergency obstetric and neonatal care
HSP: Human Subjects Protection
IDI- In: Depth Interview
LMIC: Low middle-income countries
MDG: Millennium Development Goals
N: Number
PREEMI: Preterm Resources, Education and Effective Management of Infants
SBM-R©: Standards-Based Management and Recognition
SMAG: Safe Motherhood Action Group
SSA: Sub Saharan Africa
TBA: Traditional Birth Attendant
