# Supplementary material for: Home deliveries in the capital: a qualitative exploration of barriers to institutional deliveries in peri-urban areas of Lusaka, Zambia
Source: BMC Pregnancy Childbirth. 2018 Jun 1;18:203. doi: 10.1186/s12884-018-1837-7 (PMC5984831; doi:10.1186/s12884-018-1837-7)
Supplement: Supplementary file 1 — QIA Midwife Interview Guide Final Quality improvement assessment midwife questions (DOC 40 kb) [file 12884_2018_1837_MOESM1_ESM.doc]

# Quality Improvement Assessment Midwife Interview Guide

| **Inclusion Criteria** | Exclusion criteria |
| --- | --- |
| - Qualified midwife  - Currently employed in Chawama first level hospital, Chipata clinic or George clinic | -Midwifery student  -Retired midwife |

**Demographics**

Age range: 20-30 30-40 40-50 50-60 60-70

Midwifery qualification level: Midwifery Diploma Certified midwife Degree Masters PhD

Religion:

Other qualifications:

Clinic/Hospital of Employment:

Length of stay at current employment:

Previous Clinic/Hospital of Employment:

**Personal values and Experiences**

1. Tell me about your childhood?
   1. Where you grew up. (If not explicitly mentioned ask if area is rural or urban)
   2. Who brought you up?
   3. What were you taught about labour and delivery traditionally?
2. When you were growing up was anyone in your family involved in helping pregnant women, or delivering babies?
3. Do you know if your mother delivered all her children at a health facility or at home?
4. Do you have any children of your own?
   1. How were they delivered? (Mode of delivery)
   2. Where did you deliver from? Home, health facility
   3. How would you describe your delivery experience for your last child?
5. What traditional lessons are women taught to prepare for in labour?
6. What are some of the traditional teachings about pregnancy that you know?
7. What are some of the traditional teachings about labour that you know? Do you think these traditional teachings have a positive or negative impact on labour?
8. What are some of the traditional teachings about delivery that you know? Do you think these traditional teachings have a positive or negative impact on delivery?
9. What influenced you to become a midwife?

**Personal relationships**

1. How would you describe your relationship with your current spouse/partner?

Very Good Good Fair Bad Very bad Abusive

1. How would you describe your relationship with your previous spouse/partners?

Very Good Good Fair Bad Very bad Abusive

1. How would you describe your relationships with family in your life?

Very Good Good Fair Bad Very bad Abusive

1. How would you describe your relationships with friends in your life?

Very Good Good Fair Bad Very bad Abusive

**Formal training**

1. How was what you learned in your training on labour and delivery different from what is taught traditionally?
2. How are you dealing with the differences?
3. During your training, did other midwifery students know about traditional labour and delivery beliefs that were different from what you were taught during training?
4. How did they feel about these differences?
5. Did your midwifery training include learning about cultural values/beliefs in pregnancy, labour and delivery?
6. What is the gap between what is taught in training and what happens in actual clinical practice?
7. What would help you practice better if these gaps were included in the midwifery training? **How so?**

**Professional training Vs Traditional Knowledge**

1. As a practising midwife, are there things you learnt traditionally which are helpful in your work as a midwife?
   1. If yes, what do you use in your work as a midwife?
   2. What has it helped? (For newborn babies /for mothers)
2. Are there some things you learnt during training about delivery and labour that you think are less helpful compared to what you learnt from traditional elders?
   1. What are those?
3. Are there aspects of traditional teachings about labour and delivery that you think are helpful in your nursing practice?
   1. What are those?
4. Have you ever come across other midwives practicing what is taught traditionally about labour and delivery in the field?
   1. What were those?

**Health worker Perception of mothers**

1. Do you ever have mothers in labour requesting you to allow them to apply or perform some traditional practices in the health facility ( e.g. rub some herbs or drink some herbs or tie some herb on chitenge etc.)?
   1. If yes, please share some examples
   2. How do you deal with such situations?
2. Do you ever have mothers who express that they prefer traditional approaches taught by traditional elders on labour and delivery?
   1. When did you last have such happen?
   2. What do you do if this happens?
3. What do mothers think about the medical approach to labour and delivery practiced in health facilities?

**Perceptions of relationships among pregnant women, health providers and communities**

1. How would you describe the relationship between women in this community and bana chimbusas?
2. How would you describe the relationship women in this community have with traditional birth attendants?
   1. Please give reasons for your views
3. How would you describe your current relationship as a nurse with pregnant women in this community?
4. What do mothers think about midwives at your health facility?
   1. Why do you think mothers hold these views?
   2. Do mothers in this community trust Midwives? Please explain your answers
