# Supplementary material for: Home deliveries in the capital: a qualitative exploration of barriers to institutional deliveries in peri-urban areas of Lusaka, Zambia
Source: BMC Pregnancy Childbirth. 2018 Jun 1;18:203. doi: 10.1186/s12884-018-1837-7 (PMC5984831; doi:10.1186/s12884-018-1837-7)
Supplement: Supplementary file 2 — QIA Mother’s Questionnaire Quality improvement assessment mother’s questionnaire v1.0 (DOC 112 kb) [file 12884_2018_1837_MOESM2_ESM.doc]

**Inclusion criteria:**

-Those who live in Chipata catchment area(more than 3 months)

-Delivered last child at Chipata clinic

-Home delivery in Chipata catchment area

- Child less than 2 years old

**Informed consent:**

-Do informed consent after introduction and checking inclusion criteria but before starting questions.

-For under 18s, parents/guardians must be present to also sign and listen to informed consent only. If no parents/guardians move on to someone else

-Check for literacy levels before starting by giving them consent form upside down and see if they can read one paragraph correctly

**Quality Improvement Assessment Questionnaire**

**English**: Hi, my name is __________ and I am part of the Safe Motherhood Action Group (SMAG) looking at the health of mothers, I am not sure if you have heard of them. I would like to talk to you concerning delivery and it will take about 40 minutes. I would like to hear your views on this topic. If you do not want to participate, you can say no. Likewise, if you decide to be interviewed, this is not for any rewards but you will be giving feedback that will be provided to the government on the health services being provided in your community. I will be writing what you will be telling me to make sure I do not miss out important information but will not put your names down. If you agree, I would like to use a recorder to record your responses but it will only capture your voice and not your face. Everything we discuss will be held in strict confidence.

**English:** SMAG: Are you ready to begin?

***Demographics***

**English**: SMAG: I would like to start with some basic questions about you…

What year were you born? OR How old are you?

What is your occupation?

What is your marital status?

What is your tribe?

How many children do you have?

How old were you when you had your first child? OR In what year did you have your first child?

**Section A: Screening Questions**

**English:1.** Do you have a local clinic where women can go for delivery in this community?

A. Yes

B. No

C. Don’t know/won’t say

**English: 2a.** Which clinic or hospital do people in your area deliver from?

**English: 2b:** If Chipata clinic/hospital is mentioned ask, “Do you prefer to call it a clinic or hospital?”

**English:** **3.** From your understanding, when should you use the clinic or hospital for delivery?

A. When you have a risky pregnancy

B. Everytime you are in labour(if they pick this option ask how they define labour using the following options):

| When your water breaks |  |
| --- | --- |
| When your contractions start(pain that feels like strong period pains) |  |
| Backache |  |
| When you produce some discharge after the water breaks |  |

C. Don’t know/No response

D. Other……………………………………………………………………………………………….

**English**: **4.** Can you think back to your last pregnancy? Would you be able to describe what happened and what your experience was once labour started up until you delivered your child?

a. ***If delivered at health facility:*** What made you to deliver at your clinic or hospital?

b. ***If delivered at home:*** What made you not deliver at your clinic or hospital when in labour?

c. Based on what you have told me, how likely do you think it would be for you to have a complication if you did not deliver at the clinic or hospital when in labour?

A. Very likely

B. Somewhat likely

C. Not likely at all

**If answered A or B ask which complications? Ask why for all responses(A, B or C)**

d. Based on what you have just told me, how serious do you think it would be if you did not use the clinic or hospital for delivery?

A. Very serious

B. Somewhat serious

C. Not serious at all

**Ask why.**

e. Do you know any health centre or district rules in place that make it more likely for you to use your local clinic or hospital for delivery? **If yes, what are they and why?**

f. Are there any community rules in place that you know of that make it more likely for you to use your local clinic or hospital for delivery? **If yes, what are they and why?**

g. Are there any cultural rules or taboos that you know in favour of pregnant women using the local clinic for delivery when in labour?

A. Yes

B. No

C. Don’t know / won’t say

**If yes, what are they?**

1. Has anything changed about the way you feel about using the facility for labour now, compared to back then (during your last delivery)?

**English**: **5.** Just to confirm, based on what you just told me, did you use the clinic or hospital for your last delivery?

A. Yes

B. No

**English**: **6.** You said you had X children. Can you tell me where you delivered each of those. Was it at home, at the hospital, or at the clinic? If woman has more than one child,use the grid below to ask the women to **tick** where she delivered her other children.

| Child | Home | Hospital(UTH/Levy) | Local health facility |
| --- | --- | --- | --- |
| 1 |  |  |  |
| 2 |  |  |  |
| 3 |  |  |  |
| 4 |  |  |  |
| 5 |  |  |  |
| 6 |  |  |  |

**Section A: Behaviour Questions**

***[Perceived Self-Efficacy/Skills]***

**English: 1.** Based on what you know and how you feel would you use the clinic or hospital for your next delivery?

A. Yes

B. Possibly

C. No

D. Don’t know

**PROBE: Why?**

*[****Perceived Positive Consequences]***

**English:** **2.** What do you think are or would be the advantages of delivering at the clinic or hospital when you are in labour?

**Anthing else?**

***[Perceived Negative Consequences]***

**English**: **3.** What do you think are the disadvantages of delivering at the clinic or hospital when one is in labour? (**tick options if mentioned by woman**)

|  | | Independently Listed | Mentioned when probed |
| --- | --- | --- | --- |
| Distance | Time |  |  |
|  | Cost |  |  |
| Being shouted at | |  |  |
| Paying for delivery materials(bucket, jik etc) | |  |  |
| No family support | |  |  |
| Medical staff(doctors, midwives, nurses) don’t help | |  |  |
| Against wishes of my husband | |  |  |
| Against wishes of my family | |  |  |

Other responses not included in table:

***[Perceived Social Norms]***

**English:** **4a**. Who are the people that you go to for advice when you are pregnant?

|  | Independently Listed | Mentioned when probed |
| --- | --- | --- |
| Spouse |  |  |
| Mother |  |  |
| Grandmother |  |  |
| Sisters |  |  |
| Aunties |  |  |
| Other family members |  |  |
| Friends |  |  |
| Nurses |  |  |
| Doctors |  |  |
| Neighbours |  |  |
| Church mates |  |  |
| Pastor/Priest |  |  |
| Tribal elders |  |  |
| Other: |  |  |

Anyone else?

**English:** **4b.** Among the people you go to for advice, who do you think would approve of your delivering at your local clinic for your next pregnancy?

|  | Independently Listed | Mentioned when probed |
| --- | --- | --- |
| Spouse |  |  |
| Mother |  |  |
| Grandmother |  |  |
| Sisters |  |  |
| Aunties |  |  |
| Other family members |  |  |
| Nurses |  |  |
| Doctors |  |  |
| Neighbours |  |  |
| Church mates |  |  |
| Pastor/Priest |  |  |
| Tribal elders |  |  |
| Other: |  |  |

**English:** **4c.** Among the people you go to for advice, are there any you think would disapprove of your delivering at your local clinic for your next pregnancy?

|  | Independently Listed | Mentioned when probed |
| --- | --- | --- |
| Spouse |  |  |
| Mother |  |  |
| Grandmother |  |  |
| Sisters |  |  |
| Aunties |  |  |
| Other family members |  |  |
| Nurses |  |  |
| Doctors |  |  |
| Neighbours |  |  |
| Church mates |  |  |
| Pastor/Priest |  |  |
| Tribal elders |  |  |
| Other: |  |  |

**English:** **4d.** What do you think your family thinks about you delivering at a clinic or hospital for your next pregnancy?

**English:** **4e.** What do you think your friends and neighbours think about you delivering at a clinic or hospital for your next pregnancy?

**English:** **5a.** Do you think that God would approve of you using your local clinic for delivery every time you are in labour? **[Please explain]**

**English:** **5b.** Do you think that your traditional elders would approve you using your local clinic for delivery every time you are in labour? **[Please explain]**

***[Perceived Access]***

**English:** **6.** If you were to get pregnant again, would it be difficult for you to use your local clinic to deliver when in labour? **[Please explain]**

***[Perceived Susceptibility/Perceived Risk]***

**English:** **7.** Based on what you know, how likely is it that a woman could get a complication in pregnancy during labour?

A. Very likely

B. Somewhat likely

C. Not likely at all

**If answered A or B ask which complications. Ask why for all responses(A, B or C)**
